# Individual- and Neighborhood-Level Predictors of HIV Care Continuum Progression: Clark County, Nevada

**DOI:** 10.1093/ofid/ofaf409

**Published:** 2025-07-11

**Authors:** R Goyal, A Wells, V Burris, A Stachnik, P Tang, L Collins, S R Mehta, J Dufresne, S J Little

**Affiliations:** Department of Medicine, University of California San Diego, San Diego, California, USA; Department of Medicine, University of California San Diego, San Diego, California, USA; Southern Nevada Health District, Las Vegas, Nevada, USA; Southern Nevada Health District, Las Vegas, Nevada, USA; Nevada Department of Human Services, Carson City, Nevada, USA; Nevada Department of Human Services, Carson City, Nevada, USA; Department of Medicine, University of California San Diego, San Diego, California, USA; Department of Medicine, VA Medical Center San Diego, San Diego, California, USA; Healthcare Consultant, Louisville, Colorado, USA; Department of Medicine, University of California San Diego, San Diego, California, USA

**Keywords:** HIV care continuum, late-stage diagnoses, poverty, social determinants of health, viral suppression

## Abstract

**Background:**

As the US human immunodeficiency virus (HIV) epidemic is disproportionately affecting underserved communities, it is critically important to investigate how social determinants of health affect diagnosis, treatment, and prevention of HIV infection and access to care. This article presents an investigation of the HIV epidemic in Clark County, Nevada, to identify local predictors of progression along the HIV care continuum.

**Methods:**

Deidentified HIV surveillance data from 2011 to 2022 were analyzed. We investigated associations between stages of care and individual-level demographics and zip code–level characteristics using (1) generalized linear mixed-effects models for univariate analysis, (2) penalized generalized linear mixed-effects models to simultaneously conduct variable selection and estimation for multivariate analysis, and (3) geospatial analysis.

**Results:**

Individual-level factors (diagnosis year, age at diagnosis, being Hispanic, being a man who has sex with men, sex at birth, and individual-level membership in a genetic cluster) and zip code–level factors (genetic clustering and social determinants of health, including level of poverty, proportion Hispanic, proportion with high school education, proportion white, and employment status) were associated with progression through the care continuum. A key result from our multivariate analysis is higher-poverty areas are associated with lower rates of persons with HIV in care (estimate [SE], −0.42 [0.17]; *P* = .02) and viral suppression (−0.48 [0.15]; *P* = .001).

**Conclusions:**

Our findings highlight the need for linking and engaging individuals in higher-poverty neighborhoods to medical providers. Furthermore, our results support the need for additional treatment adherence services in those same neighborhoods to increase viral suppression rates.

Efforts to combat the human immunodeficiency virus (HIV) epidemic have evolved significantly over the past decades, leading to remarkable reductions in new HIV infections [1]. While this achievement should be applauded, the epidemic in the United States is increasingly disproportionately concentrated among underserved communities [2]. Given this context, there is increased interest in investigating how social determinants of health (SDOH)—“the nonmedical conditions that influence health outcomes” [3]—affect diagnosis, treatment, and overall management of HIV infection [4–8].

The HIV care continuum, also known as the HIV treatment cascade, provides a comprehensive framework to assess the progress of individuals with HIV from diagnosis to sustained viral suppression [9]. Understanding the individual-level characteristics and broader SDOH factors that influence progression along the care continuum is crucial for developing effective interventions and optimizing HIV care delivery. Several epidemiologic studies have examined associations between SDOH and the HIV care continuum progression for large geographic regions (eg, national) [5, 10, 11]. However, a more recent systematic review has shown that investigating the unique dynamics and predictors of progression in specific geographic areas is essential as the influence of neighborhood factors on progression through the HIV care continuum varies by geographic region and the demographics of people with HIV (PWH) [12]. The majority of studies included in the review indicated that care continuum outcomes were associated with local conditions, including distance to care in Washington, DC [13], and neighborhood poverty in New York City [14]. However, several studies, including in Chicago [15], South Carolina [16], and Florida [17], did not find an association between social and economic deprivation and HIV care continuum outcomes. Furthermore, such local understanding is potentially necessary for tailoring interventions to address local challenges [18].

This article presents an analysis of predictors of progression along the HIV care continuum for Clark County, Nevada. Clark County is one of the 48 priority jurisdictions for the Ending the HIV Epidemic initiative [19]. In 2022, there were 11 518 PWH living in Clark County, resulting in a prevalence of 487 per 100 000 population [20]. In 2022, there were 21 new HIV diagnoses per 100 000 population, with a total of 488 new cases identified across the county [20]. Clark County has the highest number and rate of PWH of any county in Nevada [21], and it ranks seventh highest among US counties in terms of new HIV diagnoses rates [22].

We identified individual-level characteristics and SDOH associated with progression along the HIV care continuum in Clark County. In particular we assessed factors that could influence timely HIV diagnosis, being in care, and achievement of viral suppression. To achieve this objective, we conducted a comprehensive analysis of deidentified data collected from the Clark County Enhanced HIV/AIDS Reporting System (eHARS), an application that assists health departments with reporting, data management, analysis, and data sharing.

## METHODS

### Data

We studied 3597 PWH who had HIV diagnosed in Clark County between 2011 and 2022 and resided in Clark County on 31 December 2022. Residence is based on zip code of diagnosis and current address available in eHARS. Restricting the analysis to individuals who both received their diagnosis and lived in Clark County enables better isolation of the association between local predictors and progression along the HIV care continuum. For each individual, we analyzed deidentified demographic characteristics (such as race, transmission risk, and age), residential zip codes (at diagnosis and most recent, as of 2022), clinical laboratory values (specifically CD4^+^ T-lymphocyte count and viral load), and HIV sequence data.

These sequences were HIV-1 partial pol sequences collected for antiretroviral resistance testing in the course of routine clinical care, as ordered by clinicians in Clark County. The sequences were processed by different laboratories but eventually entered into eHARS, from which our analyses were performed. Population statistics that represent aspects of SDOH factors at the zip code level (including race, ethnicity, poverty, employment, and education) were obtained from the American Community Survey (ACS) 2021 report (5-year averages were used). Locations of HIV testing sites supported by Southern Nevada Health District (SNHD) were provided by SNHD.

All studies were reviewed and approved by the University of California San Diego Human Research Protections Program. The study conducted secondary data analysis, and no additional primary data were collected from participants. Data were anonymized before they were received from Clark County.

### Outcomes

We identified individual-level characteristics and zip code–level SDOH associated with (1) late-stage diagnosis; (2) being in care; and (3) achievement of viral suppression. HIV infection stages were classified according to the Centers for Disease Control and Prevention (CDC) surveillance case definitions, which delineate stages 0–3 based on CD4^+^ cell count. Stage 0 denotes early HIV infection, identified by a negative HIV test result within 180 days preceding a confirmed positive result [23]. Stages 1, 2, and 3 are characterized by a CD4^+^ cell counts of ≥500/µL, 200–499/µL, and <200/µL, respectively [23]. In this study, we designated an individual as having a late-stage diagnosis ​if both HIV and HIV stage 3 were diagnosed within 90 days of each other; this definition is consistent with previous research published by CDC [24].

We defined being in care as having a viral load, CD4^+^ cell count, or HIV genotype laboratory result during 2022 or the last quarter of 2021. We assumed that PWH were virally suppressed if their viral load was <200 copies/mL of blood on a visit; this definition is consistent with the metric for viral suppression used by the CDC [25]. We used the last observed viral load in the calendar year of 2022 if >1 was observed. If an individual did not have a viral load result during 2022, we included values that occurred in the last quarter of 2021. If neither of these periods included a viral load, the person was considered not virally suppressed.

### Predictors

We investigated individual-level characteristics as well as SDOH factors represented by zip code–level population characteristics. For individual-level characteristics, we investigated ethnicity and race (Hispanic of any race, black non-Hispanic, white non-Hispanic, and other non-Hispanic [eg, Asian, Native American]), whether the individual reported as a man who has sex with men or a person who injects drugs, age at HIV diagnosis, year of diagnosis, sex at birth, and whether an individual's HIV sequence is sufficiently similar to another individual in Clark County to be considered genetically linked (ie, genetically clustering with ≥1 other individual). To assess linkage, we computed genetic distances between viral sequences using the TN93 nucleotide substitution model [26]. Sufficient similarity was inferred when the genetic distance between the 2 sequences were below a distance threshold of 0.015 (ie, 1.5% sequence divergence). For our analysis, if an individual did not have a sequence, we labeled him or her as not genetically clustered.

To capture aspects of SDOH, we used the following metrics at a zip code–level based on data from the ACS data (accessed using tidycensus in R software [27]): the proportion of individuals (1) under the poverty line (neighborhood poverty), (2) of Hispanic ethnicity, (3) who completed high school, (4) of white race (any ethnicity), and (5) employed. Using the genetic (sequence data, we calculated the proportion of individuals in a zip code that were genetically linked (neighborhood genetic clustering), including linkage within or across zip codes. SDOH metrics were associated with PWH based on their residential zip code. For analyses using late-stage HIV diagnosis as the outcome, the residential zip code at the time of diagnosis was used to determine SDOH values. In contrast, for analyses with in-care status and viral suppression as outcomes, the most recent residential zip code (as of 2022) was used. For consistency across all analyses, all SDOH metrics were based on 2021 values.

The proportions for all SDOH metrics were converted to binary high and low categories such that 50% of PWH in Clark County resided in a neighborhood labeled as high or low. For example, 50% of PWH in Clark County live in a zip code labeled as high poverty. Some zip codes in the region had incomplete ACS data. We excluded a total of 117 of 3714 individuals (3.2%) who had corresponding zip codes at diagnosis or current residence where ACS metrics were incomplete. Finally, we created a binary variable for each zip code indicating whether the area had an HIV testing site supported by SNHD. For clarity, at times, we refer to zip code–level predictors as neighborhood predictors.

### Statistical Analysis

We conducted univariate and multivariate analyses. For our univariate analysis, we identified individual-level and zip code–level variables associated with our 3 outcomes by fitting generalized linear mixed-effects models (GLMMs) with a logit link function. The models were adjusted for clustering at the zip code level by using a random effect for intercept. We reported the regression coefficient (log odds ratio), SE, and *P* value from each univariate logistic regression model. We also generated geographic maps to examine the geospatial relationship of our outcomes.

For our multivariate analysis, we used GLMMs with an L1 penalty, that is LASSO (Least Absolute Shrinkage and Selection Operator) regression, to simultaneously conduct model selection and estimation [28, 29]. LASSO regression facilitates variable selection by applying a penalty term to the regression coefficients, effectively shrinking some coefficients to zero and thus achieving feature selection. To optimize the performance and find the best tuning parameter (λ), which controls the level of regularization, we adopted a cross-validation approach. Specifically, *k*-fold cross-validation was used, in which the dataset was divided into *k* = 10 subsets and each time 1 subset was held out as a validation set while the model was trained on the remaining *k* − 1 subsets. This process was repeated *k* times, and the average error was computed to determine the optimal λ whose mean prediction error was within 1 SE of the minimum mean prediction error. To estimate *P* values, a final reestimation was conducted, in which a model that includes only the variables corresponding to the nonzero fixed effects was fitted by simple Fisher scoring. Similar to the GLMMs, the LASSO models control for clustering at the zip code level using a random effect for intercept. We reported the regression coefficient (log odds ratio), SE, and *P* value. All analyses were conducted using R software (version 4.3.0).

## RESULTS

### Study Population Characteristics

There were 3597 PWH who both had HIV diagnosed and were residing in Clark County with zip code–level metrics (Table 1). The majority of them (3078 [86%]) were male. A total of 1266 (35%) were Hispanic of any race, while 1050 (29%) and 966 (27%) were black (non-Hispanic) and white (non-Hispanic), respectively. The majority of PWH (2372 [66%]) identified as men who have sex with men (MSM); however, 708 (20%) did not report any type of risk factor. The median age at HIV diagnosis was 32.8 years (range, 13.7–80.2 years). Of the study population, 1161 (32%) had ≥1 viral sequence collected. Based on our molecular analysis, 517 individuals were genetically linked (45% of those with a sequence) to ≥1 other individual in the county (Table 1).

**Table 1.** Descriptive Statistics

Characteristic	PWH, No. (%)^a^						
	Overall (n = 3597)	Early-Stage HIV Diagnosis (n = 2781)	Late-Stage HIV Diagnosis (n = 816)	Not in Care (n = 760)	In Care (n = 2837)	Unsuppressed (n = 1133)	Suppressed (n = 2464)
Individual-level predictors							
Age at diagnosis, mean (range), y	35.5 (13.7–80.2)	34.2 (13.7–77.1)	39.7 (14.9–80.2)	33.1 (14.9–69.8)	36.1 (13.7–80.2)	33.7 (14.3–76.8)	36.3 (13.7–80.2)
Year of HIV diagnosis, mean (range)^b^	2017.2 (2011–2022)	2017.3 (2011–2022)	2016.7 (2011–2022)	2016.9 (2011–2022)	2017.3 (2011–2022)	2017.0 (2011–2022)	2017.3 (2011–2022)
Race/ethnicity							
Black	1050 (29)	815 (29)	235 (29)	266 (35)	784 (28)	406 (36)	644 (26)
Hispanic	1266 (35)	980 (35)	286 (35)	258 (34)	1008 (36)	366 (32)	900 (37)
Other	315 (8.8)	242 (8.7)	73 (8.9)	57 (7.5)	258 (9.1)	84 (7.4)	231 (9.4)
White	966 (27)	744 (27)	222 (27)	179 (24)	787 (28)	277 (24)	689 (28)
Sex							
Female	519 (14)	383 (14)	136 (17)	113 (15)	406 (14)	165 (15)	354 (14)
Male	3078 (86)	2398 (86)	680 (83)	647 (85)	2431 (86)	968 (85)	2110 (86)
Transmission risk factor							
Heterosexual contact	244 (6.8)	183 (6.6)	61 (7.5)	57 (7.5)	187 (6.6)	82 (7.2)	162 (6.6)
IDU	127 (3.5)	95 (3.4)	32 (3.9)	22 (2.9)	105 (3.7)	40 (3.5)	87 (3.5)
MSM	2372 (66)	1883 (68)	489 (60)	451 (59)	1921 (68)	676 (60)	1696 (69)
MSM and IDU	140 (3.9)	111 (4.0)	29 (3.6)	32 (4.2)	108 (3.8)	48 (4.2)	92 (3.7)
No reported risk	708 (20)	504 (18)	204 (25)	197 (26)	511 (18)	285 (25)	423 (17)
Other	6 (0.2)	5 (0.2)	1 (0.1)	1 (0.1)	5 (0.2)	2 (0.2)	4 (0.2)
Sequence available						…	
No	2436 (68)	1904 (68)	532 (65)	605 (80)	1831 (65)	772 (68)	1664 (68)
Yes	1161 (32)	877 (32)	284 (35)	155 (20)	1006 (35)	361 (32)	800 (32)
Genetic cluster							
No	3080 (86)	2358 (85)	722 (88)	709 (93)	2371 (84)	991 (87)	2089 (85)
Yes	517 (14)	423 (15)	94 (12)	51 (6.7)	466 (16)	142 (13)	375 (15)
Zip code–level predictors, mean proportion mean (range)							
In cluster	0.1 (0.0–1.0)	0.1 (0.0–0.3)	0.1 (0.0–0.3)	0.1 (0.0–0.2)	0.1 (0.0–1.0)	0.1 (0.0–1.0)	0.1 (0.0–0.2)
Below poverty level	0.2 (0.0–0.3)	0.2 (0.0–0.3)	0.2 (0.0–0.3)	0.2 (0.0–0.3)	0.2 (0.0–0.3)	0.2 (0.0–0.3)	0.2 (0.0–0.3)
Hispanic ethnicity	0.4 (0.0–0.7)	0.4 (0.0–0.7)	0.4 (0.0–0.7)	0.4 (0.1–0.7)	0.4 (0.0–0.7)	0.4 (0.1–0.7)	0.4 (0.0–0.7)
High school education (or above)	0.8 (0.6–1.0)	0.8 (0.6–1.0)	0.8 (0.6–1.0)	0.8 (0.6–1.0)	0.8 (0.6–1.0)	0.8 (0.6–1.0)	0.8 (0.6–1.0)
White race	0.5 (0.3–0.9)	0.5 (0.3–0.9)	0.5 (0.3–0.9)	0.5 (0.3–0.9)	0.5 (0.3–0.9)	0.5 (0.3–0.9)	0.5 (0.3–0.9)
Employment	0.9 (0.9–1.0)	0.9 (0.9–1.0)	0.9 (0.9–1.0)	0.9 (0.9–1.0)	0.9 (0.9–1.0)	0.9 (0.9–1.0)	0.9 (0.9–1.0)

Abbreviations: HIV, human immunodeficiency virus; IDU, injection drug user; MSM, men who have sex with men; PWH, people with HIV.

^a^Data represent no. (%) of PWH unless otherwise specified.

^b^For the year of HIV diagnosis, decimals represent the fraction of the year elapsed at the time of diagnosis.

In terms of SDOH, the average neighborhood poverty rate across all PWH based on current zip code was 20%, the average neighborhood unemployment rate was 10%, and the average neighborhood proportion without a high-school diploma was 20%. Within communities where PWH were currently living, the neighborhood proportion of non-Hispanic white individuals averaged 50%, and the average neighborhood proportion of Hispanic persons was 40% (Table 1).

**Figure 1.**
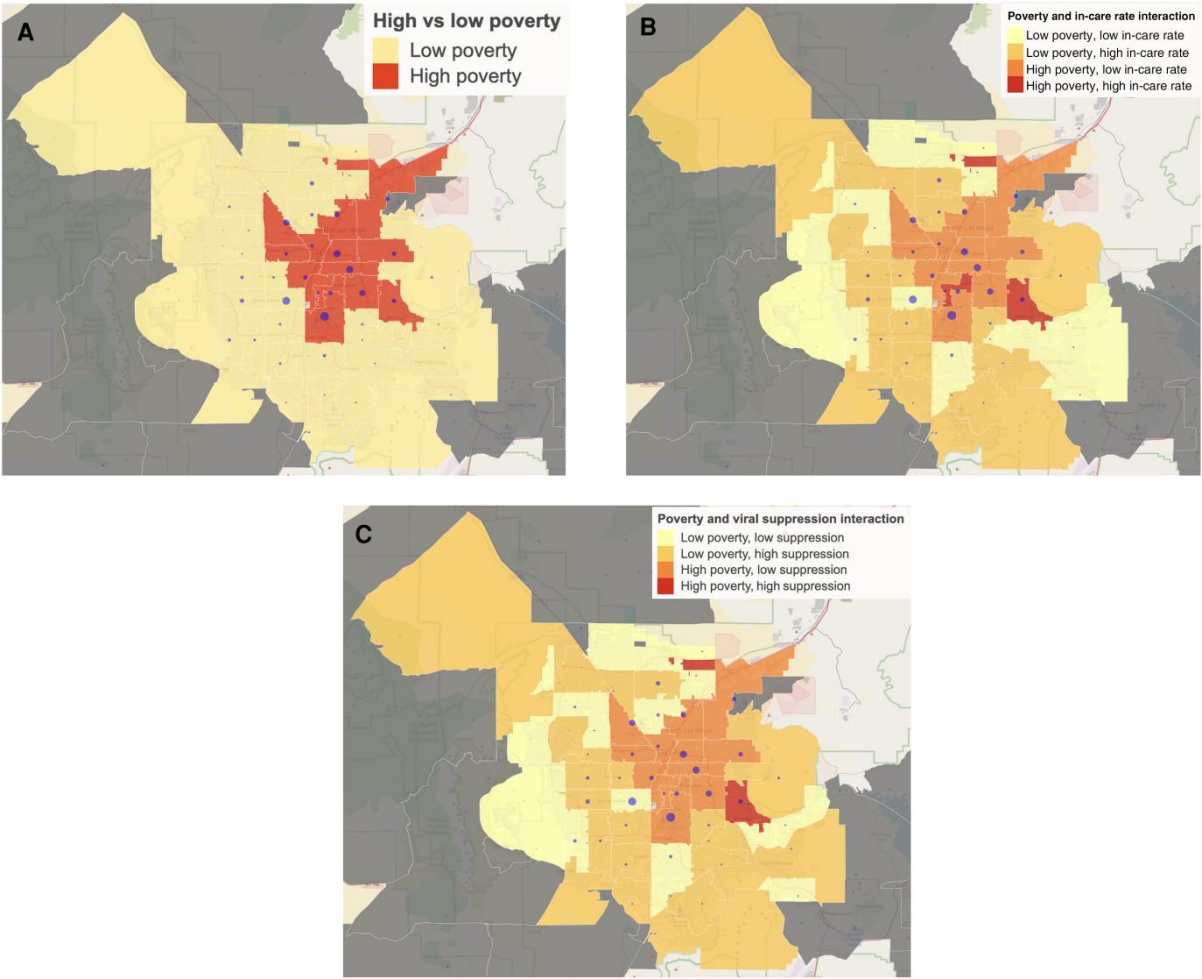
*A,* Zip codes above and below median poverty rate among people with human immunodeficiency virus (HIV; PWH). *B,* Interaction between high versus low neighborhood poverty and high versus low rates of being in HIV care. *C,* Interaction between high versus low neighborhood poverty and high versus low rates of viral suppression. Circles are proportional in size to number of PWH in the zip codes. Regions with <10 PWH were excluded from visualization.

Data visualizations of Clark County were created with zip codes colored based on whether the area has a high (*yellow*) or low (*red*) neighborhood poverty level (Figure 1*A*). As noted in Methods, we dichotomized neighborhoods such that roughly 50% of PWH are expected to reside in areas labeled as high poverty.

### Late-Stage New Diagnoses

Of the 3597 PWH, 816 (23%) had late-stage HIV diagnosed (Table 1). Both univariate and multivariate analyses indicated that individual-level demographics and SDOH were associated with having a late-stage diagnosis. Individual-level predictors of late-stage diagnosis in our univariable analysis included older age at diagnosis (estimate [SE], 0.04 [3.4 × 10^−3^]) (Table 2). Lower rates of late-stage diagnosis were associated with later year of diagnosis (estimate [SE] −0.05 [6.6 × 10^−4^]), MSM status (−0.38 [8.5 × 10^−2^]), male sex at birth (−0.22 [0.11]), and individual-level genetic clustering (−0.31 [0.12]). We did not observe any SDOH factors associated with late-stage diagnosis in the univariate models.

**Table 2.** Statistical Model Results for Late-Stage HIV Diagnosis

Predictor	PWH by HIV Stage at Diagnosis, No./Total (%)^a^		Univariate Analysis		Multivariate Analysis	
	Early Stage (n = 2781)	Late Stage (n = 816)	Estimate (SE)	*P* Value	Estimate (SE)	*P* Value
Individual-level predictors						
Year of HIV diagnosis, mean (range)^b^	2017.3 (2011–2022)	2016.7 (2011–2022)	−0.05 (6.65 × 10^−4^)	<.001^c^	−0.06 (0.013)	<.001^c^
Age at diagnosis, mean (range), y	34.2 (13.7–77.1)	39.7 (14.9–80.2)	0.04 (0.003)	<.001^c^	0.04 (0.003)	<.001^c^
Hispanic ethnicity	980/2781 (35)	286/816 (35)	−0.01 (0.085)	.92	0 (NA)	…
IDU	206/2781 (7.4)	61/816 (7.5)	0.03 (0.153)	.83	−0.1 (0.158)	.52
MSM	1994/2781 (72)	518/816 (63)	−0.38 (0.085)	<.001^c^	−0.3 (0.112)	.007^d^
Male sex at birth	2398/2781 (86)	680/816 (83)	−0.22 (0.11)	.04^e^	0.16 (0.142)	.26
Genetic link	423/2781 (15)	94/816 (12)	−0.31 (0.122)	.01^e^	−0.04 (0.13)	.78
Zip code–level predictors						
Neighborhood genetic clustering	1359/2781 (49)	375/816 (46)	−0.11 (0.104)	.31	0.01 (0.092)	.93
Neighborhood poverty level	1311/2781 (47)	370/816 (45)	−0.03 (0.108)	.76	0.23 (0.165)	.16
Hispanic ethnicity	1376/2781 (49)	368/816 (45)	−0.17 (0.103)	.09	−0.18 (0.162)	.26
Educational level	1231/2781 (44)	379/816 (46)	0.09 (0.104)	.38	−0.01 (0.173)	.96
White race	1379/2781 (50)	367/816 (45)	−0.19 (0.102)	.07	−0.27 (0.097)	.005^d^
Employment	1346/2781 (48)	434/816 (53)	0.18 (0.101)	.08	0.28 (0.133)	.03^e^
HIV testing site	1024/2781 (37)	291/816 (36)	−0.04 (0.111)	.72	−0.09 (0.092)	.30

Abbreviations: HIV, human immunodeficiency virus; IDU, injection drug user; MSM, men who have sex with men; NA, not applicable; PWH, people with HIV.

^a^Data represent no./total (%) of PWH unless otherwise specified.

^b^For the year of HIV diagnosis, decimals represent the fraction of the year elapsed at the time of diagnosis.

^c^ *P* ≤ .001.

^d^ *P* ≤ .01.

^e^ *P* ≤ .05.

Based on the multivariate analysis (Table 2), we estimated similar associations in magnitude and direction for age at diagnosis (estimate [SE] 0.04 [3.5 × 10^−3^]), year of diagnosis (−0.06 [1.3 × 10^−2^]), and MSM status (−0.30 [0.11]). However, male sex at birth and individual genetic clustering were no longer significant. For SDOH factors, we found that a higher neighborhood proportion of white (non-Hispanic) residents (estimate [SE], −0.27 [9.7 × 10^−2^]) was associated with lower rates and a higher neighborhood proportion of employment (0.28 [0.13]) with higher rates of late-stage diagnosis.

### Being in Care

Of the 3597 PWH, 2837 (79%) were identified as being in care (Table 1). Based on univariable analysis (Table 3), both individual-level demographics and SDOH were associated with being in care. Among individual-level predictors, older age at diagnosis (estimate [SE], 0.02 [3.8 × 10^−3^]), later year of diagnosis (0.04 [7.9 × 10^−4^]), MSM status (0.37 [8.7 × 10^−2^]), and individual-level genetic clustering (1.02 [0.15]) were associated with higher rates of being in care. For SDOH factors, a higher neighborhood proportion of residents with a high school diploma (or higher) in a zip code (estimate [SE], 0.19 [9.1 × 10^−2^]) was associated with higher rates of PWH being in care. A higher neighborhood proportion of residents under the poverty line in a zip code (estimate [SE], −0.27 [8.9 × 10^−2^]) was associated with lower rates of PWH in care.

**Table 3.** Statistical Model Results for Being in HIV Care

Predictor	PWH, No./Total (%)^a^		Univariate Analysis		Multivariate Analysis	
	Not in Care (n = 760)	In Care (n = 2837)	Estimate (SE)	*P* Value	Estimate (SE)	*P* Value
Individual-level predictors						
Year of HIV diagnosis, mean (range)^b^	2016.9 (2011–2022)	2017.3 (2011–2022)	0.04 (7.89 × 10^−4^)	<.001^c^	0.02 (0.013)	.13
Age at diagnosis, mean (range), y	33.1 (14.9–69.8)	36.1 (13.7–80.2)	0.02 (0.004)	<.001^c^	0.03 (0.004)	<.001^c^
Hispanic ethnicity	258/760 (34)	1008/2837 (36)	0.07 (0.087)	.41	0.13 (0.091)	.15
IDU	54/760 (7.1)	213/2837 (7.5)	0.08 (0.159)	.63	0.08 (0.165)	.63
MSM	483/760 (64)	2029/2837 (72)	0.37 (0.087)	<.001^c^	0.77 (0.113)	<.001^c^
Male sex at birth	647/760 (85)	2431/2837 (86)	0.04 (0.116)	.70	−0.52 (0.146)	<.001^c^
Genetic link	51/760 (6.7)	466/2837 (16)	1.02 (0.154)	<.001^c^	1.13 (0.16)	<.001^c^
Zip code–level predictors						
Neighborhood genetic clustering	379/760 (50)	1395/2837 (49)	−0.03 (0.096)	.77	0 (NA)	…
Neighborhood poverty	391/760 (51)	1274/2837 (45)	−0.27 (0.089)	.003^d^	−0.42 (0.172)	.02^e^
Hispanic ethnicity	388/760 (51)	1338/2837 (47)	−0.15 (0.093)	.11	0.21 (0.176)	.24
Educational level	317/760 (42)	1325/2837 (47)	0.19 (0.091)	.03^e^	0.05 (0.188)	.79
White race	356/760 (47)	1421/2837 (50)	0.13 (0.095)	.16	0.01 (0.1)	.96
Employment	353/760 (46)	1435/2837 (51)	0.16 (0.093)	.08	−0.02 (0.13)	.86
HIV testing site	285/760 (38)	989/2837 (35)	−0.09 (0.1)	.35	−0.1 (0.095)	.28

Abbreviations: HIV, human immunodeficiency virus; IDU, injection drug user; MSM, men who have sex with men; NA, not applicable; PWH, people with HIV.

^a^Data represent no./total (%) of PWH unless otherwise specified.

^b^For the year of HIV diagnosis, decimals represent the fraction of the year elapsed at the time of diagnosis.

^c^ *P* ≤ .001.

^d^ *P* ≤ .01.

^e^ *P* ≤ .05.

The multivariate analysis (Table 3) showed some slight differences. For individual-level predictors, we estimated similar associations in magnitude and direction as our univariate analyses for age at diagnosis (estimate [SE], 0.03 [4.1 × 10^−3^]), MSM status (0.77 [0.11]), and individual-level genetic clustering (1.13 [0.16]). However, year of diagnosis was no longer significant. In addition, we found that male sex at birth was associated with a lower rate of PWH being in care. For SDOH predictors, a higher neighborhood proportion of residents under poverty line (estimate [SE], −0.42 [0.17]) was significant and similar to univariate results; however, the education level of residents in a zip code was no longer significant. Therefore, neighborhood poverty level was the only significant SDOH predictor we identified, and it was inversely associated with PWH being in care.

The interaction between high and low rates of PWH in care and poverty by zip code were investigated (Figure 1*B*), almost all areas labeled as high neighborhood poverty were also below the median level of PWH being in care. Inversely, areas labeled as low poverty in the suburbs of Clark County were almost all above the median level of PWH in care.

### Viral Suppression

Of the 2837 of 3597 PWH (79%) in care, 2464 of 3597 (69%) were virally suppressed (Table 1). A total of 373 of 2837 (13%) were in care and not virally suppressed. Based on univariable analysis (Table 4), we identified the following individual-level predictors associated with higher rates of viral suppression: older age at diagnosis (estimate [SE]. 0.02 [3.3 × 10^−3^]), later year of diagnosis (0.02 [6.7 × 10^−4^), MSM status (0.40 [7.7 × 10^−2^]), being Hispanic (0.20 [7.7 × 10^−2^]), and individual-level genetic clustering (0.25 [0.11]). For SDOH predictors, high neighborhood employment rate (estimate [SE], 0.24 [8.9 × 10^−2^]) and high neighborhood proportion with a high school diploma (0.27 [8.8 × 10^−2^]) at the zip code level were associated with higher rates of viral suppression among PWH, while high neighborhood poverty rate (−0.38 [8.0 × 10^−2^]), and high neighborhood proportion of Hispanic residents in a zip code (−0.27 [8.8 × 10^−2^]) were associated lower rates of viral suppression among PWH.

**Table 4.** Statistical Model Results for Being Viral Suppressed

Predictor	PWH, No./Total (%)^a^		Univariate Analysis		Multivariate Analysis	
	Virally Unsuppressed (n = 1133)	Virally Suppressed (n = 2464)	Estimate (SE)	*P* Value	Estimate (SE)	*P* Value
Individual-level predictors						
Year of HIV diagnosis, mean (range)^b^	2017.0 (2011–2022)	2017.3 (2011–2022)	0.02 (6.7 × 10^−4^)	<.001^c^	0.02 (0.011)	.11
Age at diagnosis, mean (range), y	33.7 (14.3–76.8)	36.3 (13.7–80.2)	0.02 (0.003)	<.001^c^	0.03 (0.003)	<.001^c^
Hispanic ethnicity	366/1133 (32)	900/2464 (37)	0.2 (0.077)	.009^d^	0.22 (0.08)	.006^d^
IDU	88/1133 (7.8)	179/2464 (7.3)	−0.05 (0.137)	.71	−0.02 (0.14)	.88
MSM	724/1133 (64)	1788/2464 (73)	0.4 (0.077)	<.001^c^	0.51 (0.082)	<.001^c^
Male sex at birth	968/1133 (85)	2110/2464 (86)	0.02 (0.103)	.86	0 (NA)	…
Genetic link	142/1133 (13)	375/2464 (15)	0.25 (0.107)	.02^e^	0.3 (0.113)	.007^d^
Zip code–level predictors						
Neighborhood genetic clustering	588/1133 (52)	1186/2464 (48)	−0.15 (0.096)	.11	0.04 (0.092)	.68
Neighborhood poverty	598/1133 (53)	1067/2464 (43)	−0.38 (0.08)	<.001^c^	−0.48 (0.15)	.001^d^
Hispanic ethnicity	599/1133 (53)	1127/2464 (46)	−0.27 (0.088)	.002^d^	0.04 (0.153)	.79
Educational level	462/1133 (41)	1180/2464 (48)	0.27 (0.088)	.002^d^	−0.03 (0.166)	.85
White race	535/1133 (47)	1242/2464 (50)	0.14 (0.096)	.16	−0.02 (0.093)	.83
Employment	512/1133 (45)	1276/2464 (52)	0.24 (0.089)	.007^d^	−0.01 (0.112)	.96
HIV testing site	424/1133 (37)	850/2464 (34)	−0.1 (0.102)	.31	−0.1 (0.083)	.23

Abbreviations: HIV, human immunodeficiency virus; IDU, injection drug user; MSM, men who have sex with men; NA, not applicable; PWH, people with HIV.

^a^Data represent no./total (%) of PWH unless otherwise specified.

^b^For the year of HIV diagnosis, decimals represent the fraction of the year elapsed at the time of diagnosis.

^c^ *P* ≤ .001.

^d^ *P* ≤ .01.

^e^ *P* ≤ .05.

Based on the multivariate analysis (Table 4), we estimated similar associations in direction for viral suppression and age of diagnosis (estimate [SE], 0.03 [3.4 × 10^−3^]), MSM status (0.51 [8.2 × 10^−2^]), being Hispanic (0.22 [8.0 × 10^−2^]), individual-level genetic clustering (0.30 [0.11]), and high neighborhood poverty rate (−0.48 [0.15]). However, year of HIV diagnosis and neighborhood proportions (within zip code of residence) of Hispanic ethnicity, with a high school diploma, and employed were no longer significant. Therefore, we found that the neighborhood poverty rate was the only SDOH predictor associated with viral suppression in our multivariate analysis. The interaction between high and low rates of viral suppression and neighborhood poverty by zip code (Figure 1*C*) shows a similar pattern as being in care (Figure 1*B*).

## DISCUSSION

In summary, we found a statistically significant association with individual-level demographic characteristics for all 3 HIV care continuum steps in our multivariate analysis. In particular, MSM status was associated with improved outcomes in terms of lower rates of late-stage diagnosis, and higher probability of being in care and virally suppressed. Being older at HIV diagnosis is associated with higher probability of late-stage diagnosis; however, older age at diagnosis is associated with having a higher probability of being in care and being virally suppressed. Finally, individuals who were genetically linked have a higher probability of being in care and virally suppressed.

Regarding SDOH, we observed that neighborhood poverty was significantly associated with HIV care continuum stages in our multivariate analysis. Specifically, neighborhood poverty was associated with lower probability of being in care and viral suppression. In contrast, zip codes in the outermost regions of Clark County had below-median levels of PWH being in care even though they were low poverty; therefore, these geographic regions are areas that may benefit from additional linkage and adherence services.

The association of neighborhood poverty and poorer outcomes along the care continuum (ie, not receiving medical care and viral nonsuppression) aligns with findings of previous research [30–33] and conceptual theories of the impact of poverty on medical care [34]. Most previous research focused on community-level socioeconomic status identified an association between higher neighborhood poverty levels and increased HIV diagnosis rates [11, 32]. Research looking at HIV clinical outcomes identified an association between neighborhood poverty and increased mortality rates among PWH [33], lower CD4^+^ cell counts [31], and lower level of viral suppression [30]. Conceptual theories for these associations include limitations in transportation that may negatively affect HIV linkage to care in high-poverty areas [34]. Other studies support the association of poverty stigma with lower rates of medical visits and viral suppression [35]. Furthermore, research indicates that issues related to poverty—specifically, food insufficiency and hunger—may result in nonadherence to HIV treatment [36]. These findings collectively underscore the complex interplay between socioeconomic factors and HIV outcomes, emphasizing the need for targeted interventions to address disparities in vulnerable communities. Interventions such as universal basic income and rideshare and public transportation programs to offer transportation to HIV care visits are possible targeted interventions to reduce disparities.

Here we noted a positive association with individual-level genetic clustering and progression through the HIV care continuum. There are a few possible interpretations of this result. First, earlier diagnosis is frequently associated with a greater frequency of genetic clustering and subsequent greater linkage and engagement in care. Another possibility is that testing and sequencing are skewed to populations that are more willing to engage and participate in the health system. In this scenario, the new diagnoses that do not genetically link to others (ie, potentially presenting with later-stage HIV) might deserve evaluation of more focused strategies to link and engage these PWH in care.

Our analysis has several limitations. One limitation is that only 32% of individuals have ever had their HIV genome sequenced, which may influence estimates for the association between HIV care continuum stage and genetic clustering. We coded those without a sequence as not being clustered, thus treating individuals with a missing sequence and those with a sequence but not clustered as the same. Furthermore, not adjusting for missing sequence would likely result in lower estimates of clustering with a zip code compared with the true level of clustering. However, recent statistical advances may begin to address this challenge of missing network data [37, 38]. Another limitation is that the HIV sequences used in our analysis were only 500 base pairs; however, we do not anticipate longer sequences to substantially change our findings. In addition to these limitations, the design of our study may impede generalizing our findings to all PWH in Clark County. In particular, we restricted our analysis to individuals whose HIV was diagnosed and who are currently living in Clark County. The progression through the HIV care continuum may be different for those who move into or out of Clark County.

The findings of this study have implications for healthcare providers, policy makers, and public health agencies involved in HIV care and prevention in Clark County. By identifying the predictors that are positively and negatively associated with outcomes along the HIV care continuum, interventions can be designed to address the unique needs and barriers faced by individuals at different stages of the care continuum. Moreover, the results may inform the allocation of resources, development of focused outreach programs, and implementation of evidence-based social, structural, and prevention strategies to improve overall HIV care outcomes in the region. In particular, our findings highlight the need for linking and engaging individuals in higher-poverty neighborhoods to medical providers. Furthermore, our results support additional treatment adherence services in those same neighborhoods to increase viral suppression rates.
